# Memory load modulates graded changes in distracter filtering

**DOI:** 10.3389/fnhum.2014.01025

**Published:** 2015-01-06

**Authors:** Andria Shimi, Mark W. Woolrich, Dante Mantini, Duncan E. Astle

**Affiliations:** ^1^MRC Cognition and Brain Science UnitCambridge, UK; ^2^Department of Experimental Psychology, University of OxfordOxford, UK; ^3^Oxford Centre for Human Brain Activity, University of OxfordOxford, UK

**Keywords:** EEG, visual short term memory, attention, working memory, cognitive control mechanisms

## Abstract

Our ability to maintain small amounts of information in mind is critical for successful performance on a wide range of tasks. However, it remains unclear exactly how this maintenance is achieved. One possibility is that it is brought about using mechanisms that overlap with those used for attentional control. That is, the same mechanisms that we use to regulate and optimize our sensory processing may be recruited when we maintain information in visual short-term memory (VSTM). We aimed to test this hypothesis by exploring how distracter filtering is modified by concurrent VSTM load. We presented participants with sequences of target items, the order and location of which had to be maintained in VSTM. We also presented distracter items alongside the targets, and these distracters were graded such that they could be either very similar or dissimilar to the targets. We analyzed scalp potentials using a novel multiple regression approach, which enabled us to explore the neural mechanisms by which the participants accommodated these variable distracters on a trial-to-trial basis. Critically, the effect of distracter filtering interacted with VSTM load; the same graded changes in perceptual similarity exerted effects of a different magnitude depending upon how many items participants were already maintaining in VSTM. These data provide compelling evidence that maintaining information in VSTM recruits an overlapping set of attentional control mechanisms that are otherwise used for distracter filtering.

## Introduction

Our ability to select relevant information from our sensory input for further processing is critical for optimizing our performance on many cognitive tasks. This selection can be challenging if the information relevant for the task at hand shares sensory features with task-irrelevant information. Specifically, the challenge emerges because these two sets of input compete for representation (Desimone and Duncan, 1995). Hence, this selection requires attentional control mechanisms to enhance task-relevant inputs and/or supress task-irrelevant inputs, thereby biasing this competition in favor of relevant sensory material (Corbetta et al., 2000).

Given that our visual short-term memory (VSTM) capacity is highly limited (Cowan, 2001; Todd and Marois, 2004), encoding and maintaining only relevant information is critical for efficient performance. Indeed, the significance of filtering mechanisms for optimum VSTM performance has been well established. A number of studies have demonstrated that attentional filtering acts as a gateway mechanism for VSTM by reducing the memory load one needs to maintain and, importantly, that successful filtering is predictive of VSTM capacity (Vogel et al., 2005; McNab and Klingberg, 2008; Gazzaley, 2011). However, the reverse relationship is less well known, i.e., whether VSTM load can modulate attentional filtering during encoding and maintenance. Recent evidence suggests a more intimate relationship between attentional filtering and VSTM than has been previously shown, by documenting shared mechanisms between competition biasing and VSTM maintenance (e.g., Shimi and Astle, 2013). The behavioral evidence so far suggests that the number of items being held in memory will influence subjects’ ability to mitigate the impact of distracting stimuli. In our previous study we demonstrated that targets and distracters needed to be more perceptually distinct in order for subjects to reach asymptotic performance when VSTM was full. This behavioral result mirrors some other recent results, which show that distracter processing is attenuated when subjects are maintaining a large number of items (e.g., Rissman et al., 2009). Other studies have explored the impact of distracting stimuli on processing using a different behavioral design. For example, presenting flanking to-be-ignored stimuli alongside targets, that can be either congruent or incongruent with the target, is a good way of exploring the impact of this irrelevant information on target processing. When working memory is taxed subjects are less able to mitigate the interference from the incongruent distracters (Pratt et al., 2011). These findings are all consistent with the view that maintaining information for brief periods of time has a detrimental impact upon subjects’ ability to select targets and ignore distracters.

Here, we examined the modulatory effects of VSTM load on the attentional filtering of perceptually competing items. Distracter filtering is an ideal way of manipulating attentional control, with participants having to enhance the processing of relevant targets and supress the processing of to-be-ignored distracters. The process of filtering distracters requires participants to attenuate the activity corresponding to the representation of the distracter, and/or to enhance the activity corresponding to the representation of the target. Experiments in this area usually include trials in which there are no distracters, and average performance (and/or neural activity) is then compared with that resulting from distracter-present trials. The difference between these two trial types is then attributed to the attentional control mechanisms recruited to deal with the distracters. This difference could be in terms of neural activity, or in terms of a relative behavioral cost, such as reduced accuracy (e.g., Vogel et al., 2005). However, in reality attentional control will likely vary from moment to moment, with the control applied fluctuating in response to changing task goals or levels of potentially distracting input. Here, we explore these graded changes in attentional control. Rather than comparing distracter-present and distracter-absent trials, each trial in our task contained a number of distracters which could be variably target-like. On some trials, the distracters were perceptually very distinct from the targets, meaning that they provided little competition for representation; on other trials, distracters were perceptually more similar to the targets, thus requiring greater attentional control to bias the processing of the targets. In short, the target-distracter similarity was varied on a continuum between these two extremes, enabling us to explore the graded changes in the application of attentional control. Therefore, the analytic approach we employed in our study differs from that used in previous studies. Instead of looking at differences (either neural or behavioral) across trials with and without distracters, our strategy focuses on the graded continuous effect of distracter similarity that occurs across trials.

Research has demonstrated that attentional control mechanisms are highly flexible (e.g., Yantis and Johnston, 1990; Lavie and Tsal, 1994; Lavie, 1995) and can act not only upon sensory representations but also at later cognitive stages, upon items already stored in VSTM (e.g., Griffin and Nobre, 2003; Nobre et al., 2007; Astle et al., 2009; Gazzaley and Nobre, 2012). Whilst it is clear from these studies and those demonstrating that distracter-filtering constrains VSTM, that the spatial attention and VSTM systems can interact (e.g., Griffin and Nobre, 2003; Astle et al., 2012), the extent to which the basic functions of these two systems will trade-off against one another remains to be explored. In this study, we aimed to examine the extent to which the very process of maintaining information in VSTM would recruit those mechanisms typically used for attentional control. In our paradigm, in addition to manipulating the similarity of targets and distracters, we also varied the number of targets. This enabled us to examine memory load and attentional control effects in a single paradigm, and to track graded changes in attentional control both in terms of behavioral performance and its underlying neural processes. In particular, we sought to test the extent to which these two variables would interact, i.e., whether participants’ ability to filter distracters would change depending upon the number of items they were already holding in VSTM.

## Methods

### Participants

Fifteen healthy right-handed adults (10 female, mean age 24 ± 4.78 years SD) with normal or corrected-to-normal vision participated in the study. One participant contributed behavioral data only because of poor data quality in their EEG recording. The study was approved by the University of Cambridge Psychology Research Ethics Committee and participants provided written informed consent. Participants were recruited from the MRC Cognition and Brain Sciences Research Panel and received a monetary compensation (at a rate of £10 per hour).

### Behavioral task

**Figure 1:**
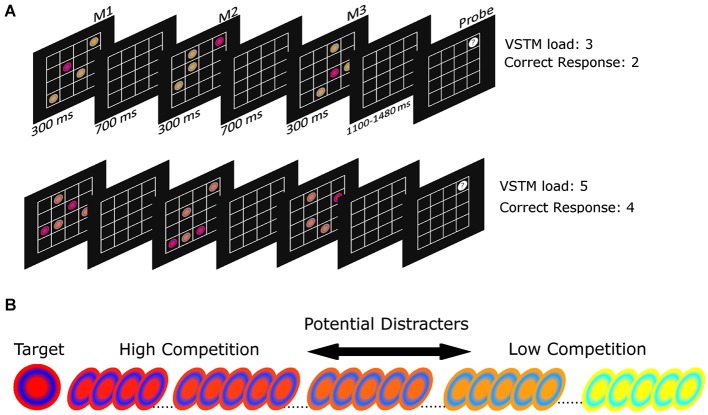
**(A)** A trial schematic showing the paradigm. Subjects are presented with three matrices of targets and distracters, which have to be remembered. Following this a single location is probed and participants must indicate in which (if any) matrix this location was occupied by a target. The top trial sequence shows a VSTM load 3 trial, in which the correct response is “matrix 2”. The lower trial sequence shows a VSTM load 5 trial, in which the correct response is “4”, meaning “no matrix”; **(B)** Examples of the target and distracter stimuli.

Figure 1 illustrates the task. On each trial, participants viewed a sequence of three matrices, each containing a target disc in a particular color (see Section *Stimuli* below). Participants were instructed to remember the location and order of the target discs in all three matrices. At the end of each trial, participants viewed a final “probe” matrix with one location highlighted; they responded as to whether a target disc had occupied the highlighted location in the preceding sequence and, if so, in which matrix the probed location had been occupied. They responded by pressing keys 1–3 on the numeric keyboard corresponding to the three matrices respectively, or key 4 if none of the previous targets had occupied the probed location. Participants were instructed to make non-speeded reaction times (RTs), and instead to attempt to maximize their accuracy.

Targets varied in number depending on the VSTM load condition: for load 3 there was one target disc in each matrix; for load 5 there were two targets in the first and second matrix followed by a single target in the third matrix. This ensured that the third matrix always contained a single target across both load conditions and therefore there was a common phase of the trial that was perceptually the same across the two levels of VSTM load (Shimi and Astle, 2013). In addition to the target disc/s, each matrix contained distracter discs. There were always three distracters per array, and these are described below.

### Stimuli

We varied the perceptual similarity between the targets and the distracters parametrically, in order to vary the difficulty of selecting targets relative to distracters (Desimone and Duncan, 1995). Each disc (0.53° in diameter) was defined in RGB space: the targets were made of a red background (R:255, G:0, B:0) with a blue ring (R:0, G:0, B:255). For each distracter we then added green in 1% increments from 1 to 255, with the most dissimilar distracter comprising a yellow background (R:255, G:255, B:0) and a cyan ring (R:0, G:255, B: 255). This was counterbalanced across participants: for half of the participants the target comprised the yellow background and cyan ring, with distracters having progressively less green. For each participant we had a target item and a set of 99 distracters, each of which was progressively more dissimilar to the target (the color of the target was consistent throughout the experiment for each participant, examples of which can be seen in Figure 1B). Each matrix comprised a 4 × 4 set of boxes, with each matrix each spanning 3.08° × 3.08°.

### Experimental design

Each matrix appeared for 300 ms and followed the previous one after 700 ms. Finally, after a randomly varied duration of 1100–1480 ms the fourth (probe) matrix appeared and remained on the screen until subjects selected their response. Following their response they briefly received feedback (the empty matrix flashed red or green for incorrect or correct responses, respectively, for 250 ms) there was then an additional 500 ms gap (with just the presentation of the empty matrix) before the start of the next trial. Participants performed 600 trials in a fully randomized order: 300 for each level of VSTM load (Load 3 and Load 5), with an equal number of 40 different levels of distracter similarity (ranging from 10% to 50% dissimilar to the target). Our previous study had demonstrated that this was a sensitive range to choose (Shimi and Astle, 2013). Participants completed 12 test blocks of 50 trials each, interleaved with self-paced breaks. We imposed the additional constraint that no location could be occupied by either a target or distracter twice on any trial, as this would introduce the situation in which subsequent items could mask or overwrite previous items. There were an equal number of trials upon which we probed a target from the first matrix (M1 trials), the second matrix (M2 trials), the third matrix (M3 trials) and trials upon which we probed a non-target location (which was always one of the distracter-occupied locations, evenly distributed across the three matrices). That is, 25% of trials were allocated to each of these four trial types. Each participant began the session with a practice block. During this block participants performed only the Load 3 condition.

### EEG acquisition

Electroencephalogram activity was recorded continuously using a BrainVision amplifier and actiCAP electrodes mounted on an elastic cap from 64 sites according to the 10–20 system. The montage included 6 midline scalp sites (Fz, Cz, CPz, Pz, POz, Oz) and 29 scalp sites over each hemisphere (FP1/FP2, AF3/AF4, AF7/AF8, F1/F2, F3/F4, F5/F6, F7/F8, FC1/FC2, FC3/FC4, FC5/FC6, FT7/FT8, FT9/FT10, C1/C2, C3/C4, C5/C6, T7/T8, CP1/CP2, CP3/CP4, CP5/CP6, TP7/TP8, TP9/TP10, P1/P2, P3/P4, P5/P6, P7/P8, PO3/PO4, PO7/PO8, PO9/PO10, O1/O2). AFz served as the ground. Blinks and eye movements were monitored with electrodes placed horizontally and vertically around the eyes. Electrode impedances were kept below 20 kΩ. We used a 250 Hz analog-to-digital sampling rate and recorded all frequencies between 0.1 and 124 Hz. The EEG was referenced online to the FCz electrode and then re-referenced off-line to the algebraic average of the left and the right mastoids. Bipolar electro-oculogram (EOG) signals were derived by computing the difference between recordings horizontal to each eye (HEOG) and between recordings vertical (VEOG) to the left eye. Participants were instructed not to move their eyes from central fixation or to blink, and any eye movements and blinks were removed using an independent component analysis (ICA): we applied a 1 Hz high-pass filter and submitted the continuous EEG to a temporal ICA (using EEGLAB; Delorme and Makeig, 2004); we correlated the time-course of each IC with our bipolar EOG channels in order to identify the ICs that corresponded to blinks and eye-movements; any that correlated >0.1 were then removed from the continuous data prior to epoching (as in Shimi and Astle, 2013). In almost all cases this resulted in two to three components being removed.

### Event-related induced response analysis

A main aim of this study was to measure the graded effect of target-distracter similarity on amplitudes and then compare it across the two levels of VSTM load. A standard ERP analysis, with its averaging procedure, is not capable of capturing these graded changes, therefore we used an *Event-related Induced Response Analysis*. We formed epochs starting 700 ms before and ending 1700 ms after the onset of the third matrix. We chose this period of the trial to form our epochs because it is perceptually equated across the two levels of VSTM load. That is, for both levels of VSTM load the third matrix contains only one target. This is important, because it means that any interaction we might observe between VSTM load and distracter-similarity represents a genuine effect of VSTM load rather than of presenting different numbers of items to be encoded. Phase and power estimates were extracted for these epochs using a continuous wavelet transform (Tallon-Baudry and Bertrand, 1999). This used six full cycles to establish the phase angles and power estimates, for frequencies between 2 and 30 Hz (in steps of 1 Hz). The power estimates were then submitted to the subsequent steps of our analysis. Because we were not interested in average evoked responses *per se*, but rather in the graded effect of target-distracter similarity, we analyzed the data using a general linear model (GLM), otherwise known as multiple regression. Within this model there were two continuous trial-wise regressors: the first was the target-distracter similarity measure (10–40% dissimilarity, inclusive) and the second was memory load (Load 3 vs. Load 5). This GLM was applied to each sample, at each electrode and for each participant. The result was a data set in which we established the linear effects of both regressors and their interaction, including their topographical distribution and time-course, within each participant. These were then fed into a group-level mixed-effects analysis, such that we identified significant effects of each regressor, or their interaction, at the population level inferred over all 14 participants.

Once the group-level analyses were completed, we identified clusters of consecutive samples (either consecutive in time and/or across neighboring electrodes). To do this, the output of the GLM was first converted into *t* statistics, using the mean of the interaction parameter across the subjects and the standard deviation of the parameter across subjects. This was repeated across all electrodes and time points to produce a single dataset that expressed our effect as *t* values. To be included in a cluster, the *t* statistic of that particular sample had to exceed 2.1. This threshold is essentially arbitrary since it is the subsequent permutation procedure that tests for significance (the same threshold is applied to each permutation to produce the null distribution). This particular clustering threshold was chosen because it approximates a two-tailed *p* = 0.05 threshold. Once our clusters were identified we recorded the size of these clusters. We then used a sign flipping permutation procedure to produce a null distribution, using 5000 permutations. With each random permutation we identified the size of any clusters where *t* > 2.1; after many permutations this resulted in a distribution that expressed the size and frequency of clusters of *t* > 2.1 that we could find by chance under the null hypothesis. We were then able to compare the size of our clusters to this null distribution, thereby identifying their relative alpha level and produce a *P* value. This approach has a number of advantages relative to more traditional approaches to significance testing with electrophysiological data: firstly, it makes no *a priori* assumptions about when or where effects are likely to be apparent within the epoch of interest, as is sometimes the case if researchers focus on particular peaks and latencies; secondly, this approach accounts for multiple comparisons over space and time, which can result in reporting spurious effects if not corrected for (Kilner, 2013). We did not enter all the GLM parameter estimates into the multiple-comparisons correction (i.e., we just explored the interaction term). This was because each additional comparison ought to be reflected in the correction; if the analysis across all time points and electrodes is being repeated multiple times to explore the effect of various regressors then these repetitions should be factored into the multiple-comparisons correction. For this reason we chose to focus on the contrast of primary interest here—the interaction between VSTM load and target-distracter similarity.

To summarize, our EEG analysis enabled us to estimate the linear effect of the continuous variable of target-distracter similarity and to compare this effect over the two different levels of load (Load 3 vs. Load 5). This is conducted over the whole set of electrodes and across all time points, without regions or time-windows of interest. The results are then fully corrected for multiple comparisons over both space and time.

## Results

### Behavioral data

Increasing the memory load significantly increased mean RT (*t*_(14)_ = 9.152, *p* < 0.001) and reduced mean accuracy (*t*_(14)_ = 7.159, *p* < 0.001). We also conducted an analysis on the accuracy of trials when the final item was probed as all trials are well matched across the two levels of VSTM load (that is the final matrix is perceptually identical across the two levels of load). We split the trials into those on which the distracters were similar or dissimilar (using a median split along the target-distracter similarity dimension). We then averaged these together, thereby reducing target-distracter similarity to a two level factor, and included it alongside VSTM Load in a 2 × 2 repeated measures ANOVA. There was a significant impact of target-distracter similarity on accuracy (*F*_(1,14)_ = 8.008, *p* = 0.013), but no significant impact of VSTM Load on the accuracy of these M3 trials (*F*_(1,14)_ = 0.096, *p* = 0.761). The interaction between these two factors approached significance (*F*_(1,14)_ = 3.521, *p* = 0.082), because there was no difference between the two levels of VSTM load when the distracters were very similar to the target (*t*_(14)_ = 0.648, *p* = 0.527), but there was when the distracters became more dissimilar (*t*_(14)_ = 3.928, *p* = 0.002).

**Figure 2:**
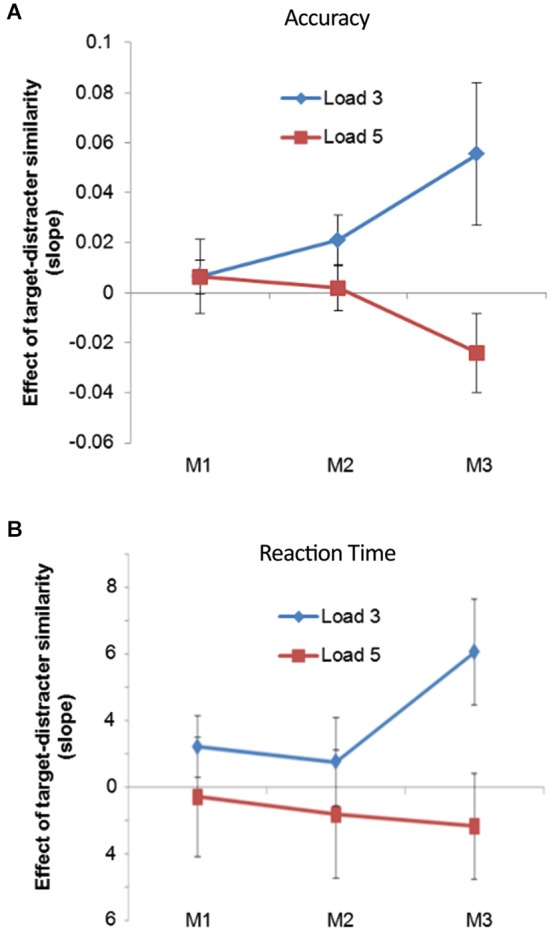
**(A)** The effect of target-distracter similarity upon accuracy produced using a regression analysis. The data are split into Load 3 and Load 5 trials, and across trial upon which the first (M1), second (M2) and third (M3) matrix was probed; **(B)** shows the same as above by with RT as the outcome measure.

However, this more conventional way of testing for the interaction between VSTM load and distracter processing is not well suited to our design, because it does not make full use of the target-distracter similarity continuum. Indeed dichotomizing our target-distracter similarity variable, something necessary for performing the conventional ANOVA, reduces the overall statistical power of the comparison (Cohen, 1983), and can produce misleading results (MacCallum et al., 2002). For this reason we also analyzed the behavioral data using a regression approach. This allowed us to include the trial-by-trial changes in target-distracter similarity, in a way that mirrored the electrophysiological analysis. We quantified the effect of target-distracter similarity on accuracy, for each trial type, using a logistic regression. The resulting slopes were submitted to a two-way ANOVA, with the within-subject factors of Order (whether the first, second, or third array was probed) and Load (Load 3 vs. Load 5). There was a main effect of Load (*F*_(1,13)_ = 11.012, *p* = 0.006), with the effect of target-distracter similarity being greatest for Load 3 relative to Load 5 trials. However, there was no main effect of serial Order (*F*_(2,26)_ = 0.128, *p* = 0.880). These two factors interacted significantly (*F*_(2,26)_ = 3.583, *p* = 0.042): memory Load had no effect upon slopes when the first item was probed (*F*_(1,13)_ < 0.001, *p* = 0.992) or when the second item was probed (*F*_(1,13)_ = 2.221, *p* = 0.160), but it did when the final item was probed (*F*_(1,13)_ = 7.964, *p* = 0.014). It is difficult to interpret the behavioral data from this task, but what can be more readily interpreted is the final simple main effect; that target-distracter similarity has a greater effect on Load 3, relative to Load 5 trials, and cannot stem from differential delay from presentation or any perceptual differences since the final array was identical across the two levels of memory load. These data can be seen in Figure 2A. The same pattern of results was apparent in the RT data, although there was no significant interaction between memory Load and serial Order (*F*_(2,28)_ = 0.975, *p* = 0.390), or significant main effects of either factor (Order: *F*_(1,14)_ = 0.510, *p* = 0.606; Load: *F*_(1,14)_ = 3.381, *p* = 0.087). These data can be seen in Figure 2B.

### Event-related induced response analysis

#### Interaction between target-distracter similarity and VSTM load

**Figure 3:**
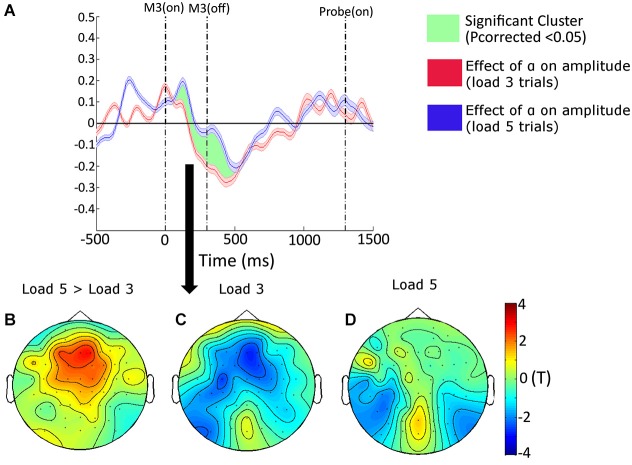
**(A)** The time-course (shown at Fz, F1, F2, F3, F4, FC1, FC2, FC3 and FC4) and topographical distribution (shown from 140 to 440 ms) of the interactive effects of target-distracter similarity, and VSTM load, on amplitudes resulting from our general linear model (GLM) analysis. The envelope of the time-course shows the standard error of the mean. The topographical plots show the difference between the target-distracter similarity effects on VSTM Load 5 and Load 3 trials, and the separate effects of this factor on amplitudes on Load 3 and Load 5 trials separately. The vertical dashed lines correspond to the onset and offset of the third matrix, and the onset of the probe display, respectively. The topography of this interaction can be seen in **panel (B)**, with the simple main effects of target-distracter similarity on load 3 and load 5 trials shown in **panels (C,D)**, respectively.

The design of our task enabled us to explore the extent to which memory load modulated the effect exerted by target-distracter similarity on amplitudes. Over the frontal and fronto-central electrodes, and from 140 to 440 ms, memory load attenuated the effect of target-distracter similarity; i.e., when memory load was high, target-distracter similarity had less of suppressive effect on power estimates (*P*_corrected_ = 0.0474). A topographical plot of this interaction can be seen in Figure 3B, with the effect of target-distracter similarity also being plotted separately for the two levels of memory load in Figure 3C and Figure 3D (Load 3 and Load 5, respectively). In Figure 3A we use the frontal (Fz, F1, F2, F3 and F4) and fronto-central (FC1, FC2, FC3 and FC4) electrodes to show the time-course of this interaction. We plotted the effect in terms of the “parameter estimate”, which corresponds directly to the relative effect of target-distracter similarity on power estimates (i.e., the steepness of the slope from our GLM). We reasoned that this reduced effect of target-distracter similarity may stem from a reduced ability to supress distracters when memory becomes full. If this were the case then we might expect that those participants who are worst at the load 5 trials would show the greatest attenuation of the target-distracter similarity effect with load. To test this we extracted the size of the interaction for each individual and correlated this alongside performance on load 5 trials. The relationship between these two factors was negative (*r* = −0.519, *p* = 0.057)—there was a tendency for the worse the participant at load 5 trials, the greater the attenuation of the distracter effect with load. Although this failed to reach significance.

**Figure 4:**
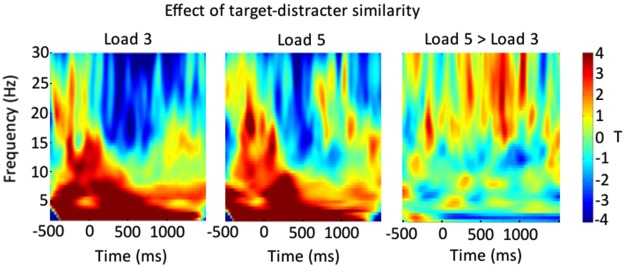
**Shows a breakdown of the result shown in Figure 3, across different time-frequency bands**. The left-most panel shows the impact of the trial-wise target-distracter similarity measure on power, in terms of a *t* statistic, for Load 3 trials. The middle panel shows this for Load 5 trials. The right-most panel shows the relative Load 5 minus Load 3 difference.

We were also interested in the frequencies that drove this suppression effect in our result. For this reason, we reanalyzed the data looking for the interaction between target-distracter similarity and VSTM load separately across different time-frequency bands (separately from 2–30 Hz in 0.5 Hz steps). The results of this can be seen in Figure 4, for Load 3 and Load 5 trials separately, and for the difference between them. From this we can see that our effect is primarily driven by a suppression of beta band activity, which is greater for Load 3 trials.

## Discussion

This study aimed to provide further insight into the relationship between the neural mechanisms of attentional selection and VSTM maintenance. To do so, we generated a set of stimuli in which we manipulated parametrically the degree of similarity between targets and distracters (from 90% similar to only 50% similar). This allowed us to vary in a continuous trial-wise manner the ease with which participants could select a sequence of targets amongst distracters. The behavioral data from this task are necessarily difficult to interpret, because on each trial there is only one opportunity to obtain a response, despite items being presented in sequence. This makes our interaction between serial order, VSTM load and our target-distracter similarity factor difficult to interpret, although we can think of a number of possible explanations: one possibility is that the impact of target-distracter similarity is swamped by a recency effect (Waugh and Norman, 1965; Shimi and Astle, 2013)—that is, performance is overall worse when the first two sets of items are probed, relative to when the final item is probed, and the impact of target-distracter similarity may only be apparent on the most accurate trials (i.e., those that come towards the end of the sequence). A second possibility is that when items are encoded into VSTM subsequent processes, such as consolidation or rehearsal, alter the attentional control effect. This could explain why the target-distracter manipulation only has a significant effect upon the final item in the sequence, presumably before these processes can take effect. A third possibility is that the target-distracter manipulation may only take effect when VSTM becomes full, towards the end of the sequence. With these data alone we do not think that we can tease apart these explanations. To do this a design is required wherein the experimenter can separate these processes, by varying sequence length and probing performance at different points in the sequence. In addition, incorporating a system of retrieval cues may enable the experimenter to separate the impact of target-distracter similarity on processing during in different phases of the trial—at encoding, during maintenance or at the point of retrieval. Nonetheless, the critical behavioral result from the current design that can unambiguously be interpreted was that the degree of similarity between targets and distracters had the greatest effect on low VSTM load trials, and that this was apparent when the final item in the sequence was probed. That target-distracter similarity has a greater effect on Load 3, relative to Load 5 trials, and cannot stem from differential delay from presentation or any perceptual differences since the final array was identical across the two levels of memory load. In order to understand the electrophysiological basis of this attention-VSTM interaction, we used a GLM in which target-distracter similarity was a continuous trial-wise regressor. As in our behavioral data, this factor interacted with VSTM load.

### Graded changes in distracter filtering are modulated by VSTM load

Our data demonstrate that distinguishing targets from distracters requires active cognitive control. The same graded changes in perceptual similarity between targets and distracters exerted effects of a different magnitude depending upon how many items participants maintained in VSTM. Following the onset of the final array of items, the more similar the targets and distracters the greater the power suppression over the frontal electrodes, primarily in the beta band. This frontal power suppression was further modulated by VSTM load; the more items being actively maintained in VSTM prior to the onset of this final item, the lesser the effect of target-distracter similarity.

A breakdown of our results shows that that this suppression is most prominent in the beta band. In general, oscillations are thought to play a critical role in coordinating activity of distinct regions of cortex and in regulating neuronal excitability (e.g., Haegens et al., 2011). However, the specific role of beta band activity is not well understood. A view growing in popularity is that whilst rapid neural rhythms indicate the integration of information over small spatial scales, slower rhythms, such as those in the beta band, correspond to the integration of information of larger spatial scales (e.g., Engel and Fries, 2010). Owing to its prominence at rest, the beta rhythm has been termed an “idling rhythm” (Pfurtscheller et al., 1996). The suppression of beta band activity has been shown to correspond closely with the implementation of voluntary top-down control processes, over both motoric and cognitive processes (see Engel and Fries, 2010, for a review). For example, coherence between frontal and parietal regions predominantly occurs within the beta-band during an endogenous top-down attentional search, but more predominantly in the gamma band during attentional pop-out searches (Buschman and Miller, 2007).

One possible interpretation of our result is that the power suppression that we observed reflects the actual processing of the distracters themselves. When VSTM load is high, it is possible that there are no more resources available for processing distracters, hence their reduced effect on power (Lavie, 1995). However, we think it is more likely that this frontal power suppression reflects participants’ top-down control of the target-distracter competition; i.e., when VSTM load was high, participants were not able to exert the control necessary to mitigate the influence of the distracters, and this is why the neural effect of the distracters is attenuated in the high VSTM load condition. We believe that our results are more readily explained by this latter interpretation. Firstly, this explanation fits well with the behavioral data. Secondly, this is in line with findings from the functional magnetic resonance imaging (fMRI) literature, which have demonstrated that when memory load is high, participants are less able to attenuate the sensory processing of task-irrelevant distracters (Rissman et al., 2009; Kelley and Lavie, 2011). These studies have shown that the active suppression of salient incongruent distracters is impaired by a concurrent maintenance task. For example, Rissman et al. (2009) showed that participants were less able to use attentional control to attenuate the processing of irrelevant visual distracters when maintaining a high load of auditory memory items. Similarly, Kelley and Lavie (2011) demonstrated that the early visual processing of distracters was modulated by short-term memory load. When memory load was high, the distracters exerted a larger effect on early sensory processing. A final example that supports this interpretation is that when memory load is high, functional connectivity between areas in frontal cortex and areas in occipital cortex is reduced (Soto et al., 2012), which may provide the underlying mechanism by which top-down control can be exerted on visual sensory processing. These results collectively suggest that resources expended in maintaining information are also used for attentional control: that is, when resources are already tied up with maintenance, attentional control functions are impaired. The experimental and analytic approach we employed here allowed us to demonstrate that graded changes in target-distracter discrimination are modulated by VSTM load. In a previous study we used behavioral measures to demonstrate this effect (Shimi and Astle, 2013); in behavioral terms, larger differences between targets and distracters were needed for successful target selection when VSTM load was high. Here, we extend this finding to demonstrate its neural basis.

An alternative explanation for our results could be that the event-related induced response effect simply reflects generic difficulty *per se*; that is, when difficulty is also high due to VSTM load, the *relative* effect of sensory discrimination difficulty is reduced. Although such a possibility exists, we believe that if this were true then we should also expect to see an impact of VSTM load on those trials when the final item is probed. However, whilst the relative effect of target-distracter similarity is altered, there is no main effect of VSTM load on these trials. A further possible interpretation could be that as distracters become more target-like, participants mistake them for targets and thus they exert a VSTM load effect. Of course this is possible and it is difficult to rule out, nonetheless, we do not think that it can account for, or undermine, the particular interaction that we report here. The same number of distracters were present across the two levels of VSTM load, so if participants began to erroneously store distracters as targets, then this should have increased the number of stored items equally for each level of VSTM load.

In the current design we defined our targets and distracters in RGB space, and did so universally for all subjects. However, in reality there is unlikely to be a linear relationship between RGB space and subjects’ perception of color. Future studies could explore the relationship between VSTM load and perceptual competition effects more sensitively by titrating these discrimination values individually for each subject. Furthermore, this psychometric process would be better implemented using a color space that better reflects the color-opponent processes of human vision, such as the LAB system (with L corresponding the lightness, and the other values corresponding to the two color-opponent channels).

In conclusion, findings here suggest that the memory load maintained in VSTM modulates graded changes in perceptual processing. These findings provide further insight to the close coupling between attentional filtering and VSTM maintenance and demonstrate that these two cognitive processes may share some underlying neurophysiological mechanisms. In short, when items are maintained in VSTM our ability to use attentional control mechanisms to distinguish targets and distracters is modulated.

## Conflict of interest statement

The authors declare that the research was conducted in the absence of any commercial or financial relationships that could be construed as a potential conflict of interest.
